# Comprehensive Evaluation of a Mucoadhesive Self-Emulsifying Anhydrous Base for Vaginal Drug Delivery

**DOI:** 10.3390/ph19040585

**Published:** 2026-04-07

**Authors:** Guiyun Song, Yi Liu, Kendice Ip, Ashley Shan, Christine Vu, Kateryna Khokhlova, Oleksandr Zdoryk, Maria Carvalho, Daniel Banov

**Affiliations:** 1 Professional Compounding Centers of America (PCCA), Houston, TX 77099, USA; 2 Department of Technology of Drugs, National University of Pharmacy, H. Skovorody Str. 53, 61002 Kharkiv, Ukraine; 3 Department of Pharmaceutical Technologies and Medicines Quality Assurance, Institute of the Professional Skills Improvement in the Field of Pharmacy, National University of Pharmacy, H. Skovorody Str. 53, 61002 Kharkiv, Ukraine

**Keywords:** vaginal drug delivery, mucoadhesive properties, self-emulsifying drug delivery systems, compounded formulations, bioadhesion testing

## Abstract

**Background/Objectives**: Compounded vaginal creams are widely used for conditions such as hormone replacement therapy, vaginal dryness, low libido, vaginal infections, etc. Recent research highlights the potential of using anhydrous bases to extend shelf life, particularly when combined with self-emulsifying and mucoadhesive properties that improve mucosal retention and enhance drug bioavailability. This study provides in vitro and ex vivo evaluation of an anhydrous vaginal base. **Methods**: Key quality indicators such as irritation potential, leakage potential, pH compatibility, mucoadhesion, and self-emulsification were assessed using the chorioallantoic membrane Hen’s Egg Test, MTT assay, texture analysis, and fluorescence microscopy. **Results**: The anhydrous vaginal base demonstrated high cell viability (>78%) and non-irritant potential (IS = 2.5) in in vitro assays. It maintained physiological vaginal pH (4.56 ± 0.05), showed strong mucoadhesive properties comparable to commercial products, and exhibited minimal leakage. Ex vivo studies confirmed its prolonged retention on vaginal tissues. The anhydrous vaginal base formed stable emulsions upon contact with vaginal fluid simulant, effectively distributing both lipophilic and hydrophilic compounds. **Conclusions**: Compared to water-containing bases, an anhydrous vaginal base shows advantages: longer retention time and lower leakage; adaptability to varying vaginal fluid levels; and efficient dispersion of both hydrophilic and lipophilic active pharmaceutical ingredients. These features support its potential use in compounded vaginal products, minimizing stability risks and enhancing patient compliance and therapeutic outcomes.

## 1. Introduction

Pharmaceutical compounding offers a flexible approach to treating various gynecological conditions and supporting women’s health by allowing healthcare providers to tailor formulations to meet specific patient needs. The ability to customize oral, topical, injectable, rectal, and vaginal dosage forms, their concentration, and combinations of active pharmaceutical ingredients (APIs) is particularly important [1]. According to current trends in pharmacy compounding for women’s health [2], the most common indications include hormone replacement therapy [3], vaginal dryness [4], low libido [5], vulvodynia [6], and vulvovaginal infections [7]. Vaginal dosage forms, including creams, play a traditional role in the treatment of gynecological diseases due to their ability to deliver APIs directly to the site of action [8]. This targeted delivery is especially important in the treatment of conditions where localized treatment can offer significant therapeutic benefits [9]. Clinical indications for prescribing compounded vaginal creams include pruritus/itching, lichen sclerosis, desquamative inflammatory vaginitis [10], decreased sex drive, radiation therapy inflammation, vaginal atrophy, vaginal and pelvic pain, candidiasis, human papillomavirus [11], radiation-induced vaginal stenosis, and dysbiosis of vaginal microbiome [12], among others.

The selection of APIs for vaginal delivery depends on the patient’s therapeutic needs and the absence of commercially available formulations. Compounding pharmacists face the complex task of combining different APIs and excipients while ensuring compatibility [13,14] and the physical and chemical stability of vaginal creams [15,16]. To ensure the best possible treatment outcomes, they also need to meet efficacy and safety standards. The composition of compounded vaginal creams often includes APIs from various pharmacological groups [4,6,17,18,19]: hormones (estriol, testosterone, estradiol, progesterone, dehydroepiandrosterone, and hydrocortisone); anesthetics (ketamine hydrochloride, lidocaine hydrochloride, and tetracaine); corticosteroids (triamcinolone acetonide and clobetasol propionate); antihistamines (ketotifen); immunosuppressants (tacrolimus); natural soothing agent (aloe vera); antimicrobials and antifungals (mupirocin, nystatin, clotrimazole, clindamycin, and boric acid); chelating agents (edetate disodium); muscle relaxants (baclofen and diazepam); anticonvulsants (gabapentin and phenytoin); bronchodilators/phosphodiesterase inhibitors (theophylline); phosphodiesterase inhibitors (sildenafil); nonessential amino acid (arginine hydrochloride); calcium channel blockers/vasodilators (nifedipine); antidepressants/analgesics for neuropathic pain (amitriptyline hydrochloride); opioid receptor antagonist (naltrexone hydrochloride).

### In Vitro and Ex Vivo Evaluation of Key Performance Parameters of Vaginal Semisolid Pharmaceutical Formulations

Promising areas for the development of compounded vaginal creams include the creation of non-irritating anhydrous bases with self-emulsifying and mucoadhesive properties. Such formulations can enhance bioavailability and minimize irritation, ensuring patient compliance. Properties of vaginal formulations are usually evaluated with a range of methods, including technological, in vitro, ex vivo, and in vivo techniques [20,21].

Non-animal in vitro methods encompass cell-based assays (human-derived vaginal epithelial and dendritic cells) and are frequently utilized to evaluate the potential for irritation and cytotoxicity (the MTT assay (3-[4,5-dimethylthiazol-2-yl]-2,5-diphenyltetrazolium bromide) and the neutral red uptake (NRU) assay) [22,23].

The Hen’s Egg Test-Chorioallantoic Membrane (HET-CAM) assay is an in vitro alternative to the traditional in vivo Draize rabbit eye irritation test and assesses the level of hemorrhage, lysis, and coagulation, indicating the potential for irritation of the test product [20]. Incubated Hen’s Egg Tests do not conflict with animal protection laws [24] and are recommended by the Interagency Coordinating Committee on the Validation of Alternative Methods (ICCVAM) [24,25].

Premature leakage, driven by human vaginal fluid (HVF) clearance, significantly reduces the residence time of the drug on the mucosal surface, ultimately leading to subtherapeutic outcomes [26]. It is therefore the task of contemporary developments to improve the coating on the vaginal mucosa, enhance drug penetration, and slow leakage [27]. To address this issue, increasing mucoadhesion in vaginal formulations is a key strategy [28,29]. In vitro leakage testing evaluates the ability of undiluted and HVF-diluted formulations to remain on an inclined surface, thus assessing their potential in vivo retention. Bioadhesion, specifically, refers to the ability of a formulation to adhere to biological tissues, and it is commonly measured using texture analyzers by determining the tensile force or work of adhesion [30,31,32,33].

In normal conditions, the vagina has a unique microbiota and is covered by a thin layer of HVF, which maintains the internal physical and chemical environment and are indicators of a woman’s health [19]. HFV pH is typically mildly acidic and crucial for the proper function of the vaginal mucosa and protection against infections [19,34,35]. Therefore, an evaluation of the effect of vaginal drug formulations on the pH of vaginal fluid is conducted, typically using a vaginal fluid simulant (VFS) [36,37].

Vaginal dosage forms with self-emulsifying properties improve drug solubility and bioavailability by spontaneously forming emulsions upon contact with HVF [38,39]. This mechanism enhances spreadability and penetration into target areas of the female reproductive tract [40]. Light and fluorescence microscopy are used to assess particle size and API distribution in the resulting emulsion [41].

The aim of this article is to provide a comprehensive in vitro and ex vivo evaluation of the key performance characteristics of an anhydrous vaginal base, focusing on its mucoadhesion, self-emulsification, and irritation potential.

## 2. Results

### 2.1. Human Vaginal–Ectocervical Tissue Viability MTT Assay

**Figure 1:**
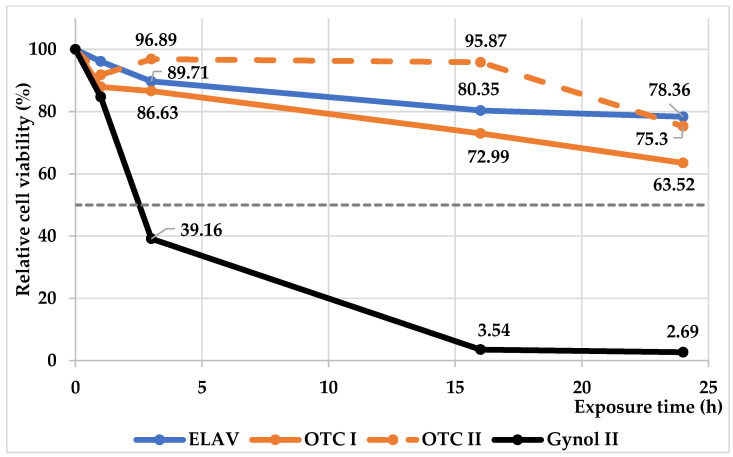
Cell viability of the EpiVaginal™ human vaginal–ectocervical tissue model following exposure to ELAV base, OTC vaginal lubricants (OTC I and OTC II), and Gynol II spermicide over time. The blue solid line represents ELAV base; the black solid line represents Gynol II; the orange solid and dashed lines represent OTC I and OTC II lubricants, respectively. ET_50_ values were determined as the exposure time required to reduce tissue viability to 50%.

At the initial time point (exposure time = 0), all samples showed 100% cell viability. As exposure time increased, as expected, a significant decrease in cell viability was observed for the positive control (Gynol II), with cell viability dropping to less than 5% after 16 h. By 24 h, cell viability of the positive control was further reduced to 2.69%, indicating high toxicity. All other samples did not reach the toxic exposure time (ET_50_) threshold within a day. After 24 h, the Ellage^®^ Anhydrous Vaginal (ELAV) base maintained 78.36% cell viability, while Over-The-Counter (OTC) I and II vaginal lubricants maintained 63.52% and 75.3%, respectively. Thus, the ET_50_ for Gynol II was approximately 3 h, whereas for the ELAV base and both OTC lubricants, the ET_50_ was greater than 24 h. The results of relative cell viability are shown in Figure 1 and in Table S1.

### 2.2. Hen’s Egg Test-Chorioallantoic Membrane Assay

**Figure 2:**
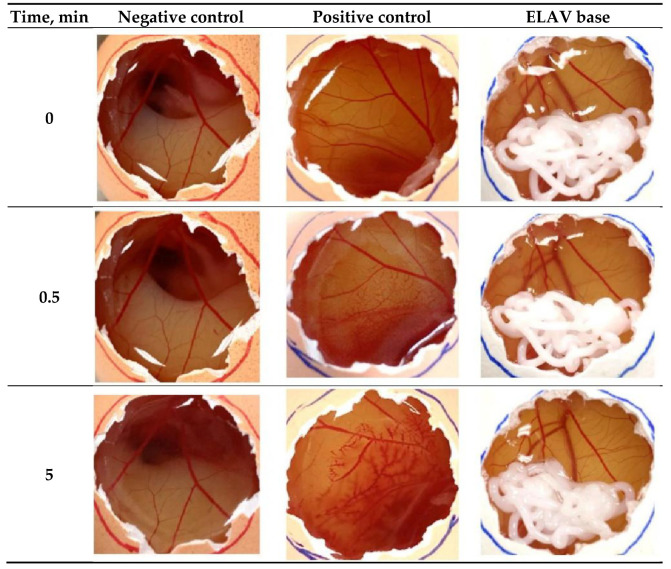
HET-CAM assay evaluating the irritation potential of ELAV base compared with 0.9% NaCl (negative control), and 0.1 N NaOH (positive control) during 5 min. The CAM was visually examined around the edges of the applied material and after gentle removal of the non-transparent formulation to ensure clear observation of vascular endpoints. No irritation reactions were observed for the ELAV base or the negative control.

In the preliminary study, both the ELAV base and 0.9% NaCl showed no irritation potential, with an irritation score (IS) of 0.00. In contrast, 0.1 N NaOH was highly irritative, with an IS of 17.00, as evidenced by lysis, hemorrhage, and coagulation (Figure 2). The CAM for test formulation was visually examined both around the edges of the sample and after gentle removal of the formulation to ensure clear observation of vascular endpoints. These findings were consistent even when the experiment was extended for 20 min, in which the ELAV base continued to show no irritation. In the outsourced study at CPT^SM^, similar results were obtained. The ELAV base demonstrated an IS of 2.50, which falls within the non-irritant range (IS 0–4.9) [42]. The cosmetic gels used as negative controls also exhibited low irritation, with IS values of 3.0 (Nivea Visage) and 2.0 (Pond’s Eye Gel).

### 2.3. Effect of the Base on the pH of HVF

**Figure 3:**
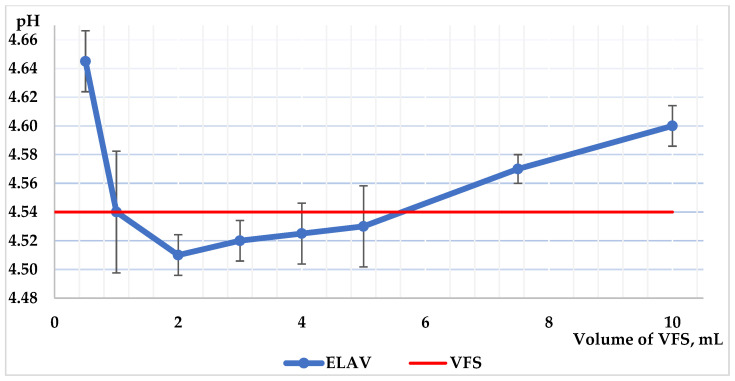
Effect of the ELAV base on the pH of VFS. The blue line represents the measured pH of the ELAV–VFS mixture, while the red line indicates the baseline pH range of VFS. Data represent mean values (*n* = 3).

The pH measurements obtained showed minimal deviation from the baseline of 4.54 for all compounded bases. All tested bases (ELAV, VersaBase^®^ Cream Compounding Base (VBC), MucoLox™ (ML)/VersaBase Gel (VBG) (50:50), OTC moisturizer) were found to maintain the mildly acidic environment of the VFS, comparable to the baseline (Table S2, Figure 3). For ELAV, the pH ranged from 4.51 to 4.65 and remained within the VFS pH range (4.54 ± 0.06) from the addition of 1 mL up to at least 8 mL. VBC showed a pH range of 4.54 to 4.94, with a higher initial pH value. ML/VBG (50:50) showed slight variations in pH values between 4.47 and 4.68. The OTC vaginal moisturizer also showed no significant effect on pH, with a range of 4.60 to 4.71.

### 2.4. Evaluation of the Mucoadhesive Properties

**Figure 4:**
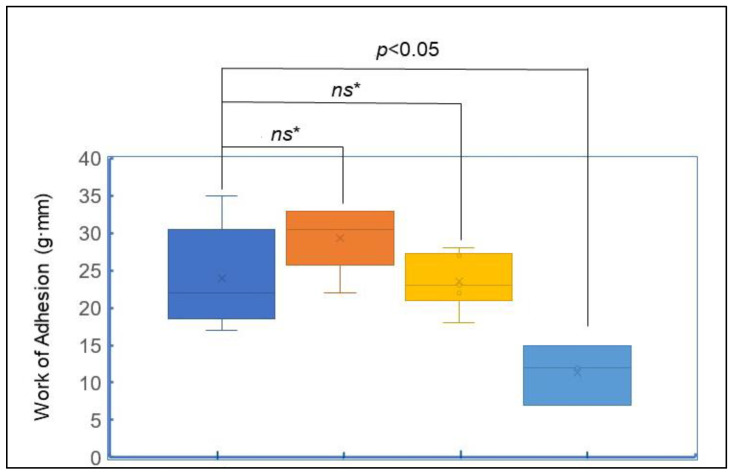
Work of adhesion determined by ex vivo mucoadhesion testing using a texture analyzer (*n* = 6). Box plots show the bioadhesive performance of the ELAV base (blue), ML/VBG (50:50) (orange), OTC vaginal moisturizer (yellow), and the negative control VFS (light blue). Each box represents the interquartile range (IQR), with whiskers indicating the minimum and maximum values. The horizontal line within each box represents the median, and the cross indicates the mean value. Statistical comparison between the tested formulations showed no significant differences (ns*, not statistically significant).

Ex vivo bioadhesion testing using a texture analyzer. The results showed that all three formulations demonstrated similar bioadhesion profiles, with the following average work-of-adhesion values (mean ± SD): ELAV base: 24 ± 6.78 g·mm, ML/VBG (50:50): 24 ± 3.62 g·mm, and the OTC vaginal moisturizer: 29 ± 4.23 g·mm (Figure 4). The differences between these results were not statistically significant, indicating that the ELAV base had a bioadhesion profile comparable to both ML/VBG (50:50) and the OTC vaginal moisturizer. The negative control (VFS alone) exhibited a significantly lower work of adhesion (11 ± 4.04 g·mm).

**Figure 5:**
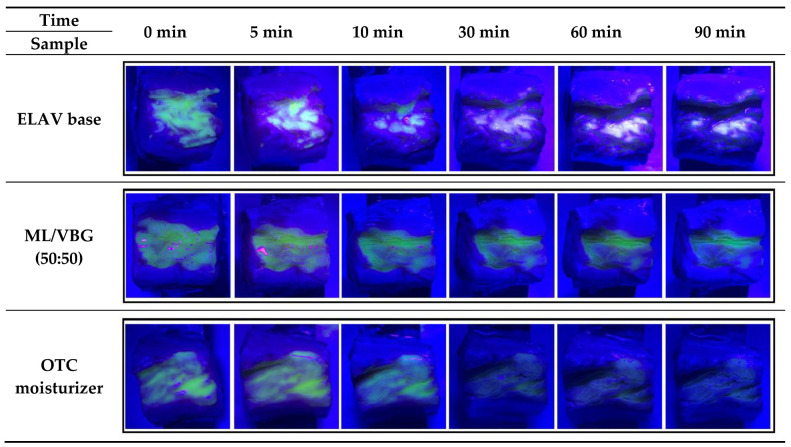
Ex vivo evaluation of mucoadhesion on porcine vaginal tissue over 90 min. Test formulations—ELAV base, ML/VBG (50:50), and an OTC vaginal moisturizer—were applied to the epithelial surface of porcine vaginal tissue and rinsed intermittently with VFS to mimic physiological conditions. Fluorescein sodium was incorporated into the formulations to visualize retention on the tissue surface under UV illumination. Images were captured at the indicated time points (0–90 min). Rows correspond to the tested formulations, while columns represent the observation time points. Persistent fluorescence indicates stronger retention of the formulation on the tissue surface.

Ex vivo testing on porcine vaginal tissues. The ML/VBG (50:50) showed strong mucoadhesion throughout the 90 min study period, with only minor fading of fluorescence (Figure 5). In contrast, the OTC vaginal moisturizer showed significantly reduced fluorescence at the end of this study, suggesting a lower retention potential. Sodium fluorescein dye was effective in visualizing the mucoadhesive properties of both ML/VBG (50:50) and the OTC vaginal moisturizer, as they are both transparent formulations. The fluorescent dye was added to all three test products to ensure that the ex vivo tissues were exposed to identical experimental conditions, despite the ELAV base being white in color and not requiring the dye for visualization (Figure S1). As the ELAV base is an anhydrous formulation and fluorescein sodium is a water-soluble compound, it was expected that the dye would be washed away by VFS, causing the fluorescence to fade. Nevertheless, the white color of the ELAV base remained clearly visible at all time points for both the test and control samples, as confirmed by the images in Figure 5.

### 2.5. Evaluation of the Leakage Potential

**Figure 6:**
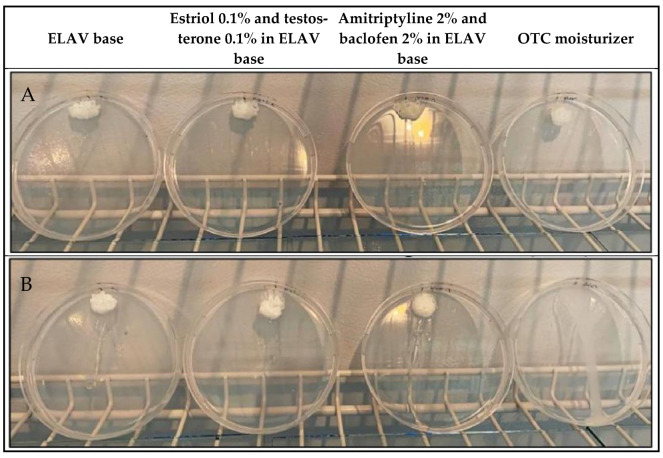
In vitro leakage test performed on agar plates inclined at 60°. Test samples included ELAV base, estriol 0.1%, and testosterone 0.1% in ELAV base; amitriptyline 2% and baclofen 2% in ELAV base; and an OTC long-acting vaginal moisturizer. (**A**) Undiluted formulations. (**B**) Formulations diluted with VFS. Images illustrate the migration behavior and leakage potential of the tested samples.

The undiluted samples exhibited no significant change in migration distance and demonstrated no movement on the agar plates, maintaining their position for the duration of the test (Figure 6A). The running speed of all samples did not exceed 0.3 mm/s, indicating high retention and minimal leakage. The diluted samples of ELAV and its formulated products also demonstrated minimal movement, with an insignificant running speed of less than 0.4 mm/s. In contrast, the OTC vaginal moisturizer exhibited a notable degree of leakage, with a displacement of 10 cm within 10 s, indicating considerable leakage potential and an average running speed of 14.44 ± 1.89 mm/s (Figure 6B).

### 2.6. Self-Emulsifying Properties

Even the naked-eye evaluation showed that the ELAV base has distinct self-emulsification properties upon gentle mixing with VFS, which distinguishes it from VBC and the Plasticized Compounding Base (PCB) (Figure S2). After mixing with VFS and incubation at physiological temperature (37 °C), ELAV spontaneously formed an emulsion, the droplet size of which gradually decreased over time. This self-emulsification phenomenon was not observed in either VBC, which maintained a stable droplet size, or PCB, which did not emulsify due to its anhydrous gel form.

However, the ELAV and VBC emulsions behaved differently under microscopic observation for 5 min after VFS addition. A conventional emulsifying base, VBC, formed a homogenous emulsion with VFS, with droplet sizes of approximately 10 μm in diameter, and it remained stable. The ELAV base not only formed droplets but also displayed a reduction in droplet size over a period of 1 to 5 min (Figure S3). PCB remained phase-separated and did not form any droplets, indicating no emulsifying behavior. When APIs (amitriptyline 2% and baclofen 2%) were incorporated into the ELAV base, similar self-emulsifying behavior was observed, with droplet formation and size reduction proceeding as observed in the API-free formulation.

The self-emulsifying behavior of the ELAV base was evident even when diluted with varying proportions of VFS (9:1, 3:1, and 1:1 ratios to VFS). Droplet formation was observed in all three ratios, underscoring the robustness of its emulsifying mechanism across a range of dilution levels (Figure S4).

**Figure 7:**
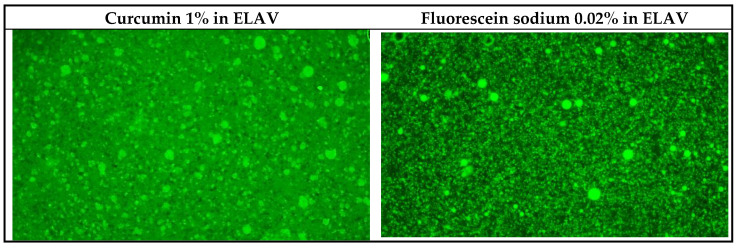
Fluorescence microscopy of ELAV base containing curcumin (lipophilic) and fluorescein sodium (hydrophilic) under green fluorescent light. The images illustrate the distribution of lipophilic and hydrophilic substances within the emulsion formed after mixing the ELAV base with VFS (100×).

The fluorescence microscopy results showed effective distribution of both lipophilic and hydrophilic substances in the ELAV base. When fluorescein sodium and curcumin were incorporated into ELAV and mixed for 2 min, each was evenly distributed and showed uniform fluorescence. After gently mixing ELAV with VFS in a 1:2 (*w*/*v*) ratio and incubating at 37 °C, the self-emulsification process was visually confirmed. Under UV light, it was clearly visible that curcumin (lipophilic) was encapsulated within the lipid droplets of the emulsion while fluorescein sodium (hydrophilic) remained in the surrounding aqueous phase. This separation within the emulsion, as shown in Figure 7 (green fluorescent light) and Figure S5 (white and green fluorescent light), highlights the efficiency of the ELAV self-emulsification process.

## 3. Discussion

### 3.1. Cytotoxicity and Irritation Characteristics

The MTT assay results demonstrate that the ELAV base and OTC I and II lubricants have a favorable toxicological profile, with a significant level of cell viability after prolonged exposure compared to the positive control. The obtained results are consistent with the approval requirements for OTC lubricants and confirm their non-toxicity to human vaginal–ectocervical (VEC) tissues. The ET_50_ values indicate that the ELAV base is non-toxic (comparable to well-characterized and approved OTC lubricants) and is expected to be safely applied to the vaginal mucosa without causing cellular damage, making it suitable for vaginal applications.

The results of both HET-CAM assays show that the ELAV base has no significant irritation potential, as demonstrated by an IS of 0 and 2.50. These scores fall well below the irritation threshold (IS > 5). The obtained data correlate with the IS of industrially manufactured creams and also align with the results from the tissue viability MTT assay.

### 3.2. pH Compatibility

The results indicate that none of the bases, including ELAV, VBC, ML/VBG (50:50), or the OTC moisturizer, significantly altered the pH of the VFS. The initial pH results for VBC correlate with the values in the study of the physical and chemical stability of 0.25 mg/g estriol and 10 mg/g vaginal creams [16], as well as the pH values of ML and VBG [43,44] and the pH limits for the OTC moisturizer stated by the manufacturer. Given that the ELAV base does not significantly alter VFS’s pH, it appears suitable for formulating vaginal preparations without compromising the physiological environment, making it a promising option for compounders delivering APIs.

### 3.3. Mucoadhesive Characteristics

During ex vivo bioadhesion testing using a texture analyzer, all experimental parameters were standardized, including contact force, contact time, sample temperature, and withdrawal speed, except for the biological variability in the tissue sample area. The standard deviations observed in the results likely stem from biological variations in the excised porcine epithelial tissues, which is a common limitation in ex vivo studies [45]. Despite this, the method proved to be a reliable and reproducible tool for evaluating mucoadhesive properties. The observed work-of-adhesion values suggest that all three samples can adhere effectively to the vaginal mucosa, potentially extending the residence time of the formulations. The bioadhesion testing results indicate that the ELAV base exhibits a strong mucoadhesive profile, comparable to the well-established combination of ML/VBG (50:50) and a marketed OTC vaginal moisturizer.

Ex vivo testing of mucoadhesive properties on porcine vaginal tissues shows that the ELAV base and ML/VBG (50:50) have similarly strong mucoadhesive profiles. The observed mucoadhesive properties of the ELAV base further support its potential suitability for use in extended-release vaginal formulations.

The results of bioadhesion tests using a texture analyzer correlate well with the ex vivo tests on porcine vaginal tissue and show that the ELAV base has a strong mucoadhesive profile, comparable to the well-established combination of ML/VBG (50:50). Despite the use of porcine tissue as a model for human vaginal mucosa, which may not fully replicate human physiological conditions, this study provides valuable insights into the mucoadhesive performance of the tested formulations.

The results of the in vitro leakage test illustrate the distinction between the properties of the ELAV base, its compounded formulations, and the OTC vaginal moisturizer. While traditional vaginal formulations, such as the OTC moisturizer included in our test, gels [46], and creams [47], often become diluted upon contact with vaginal fluids, which can result in increased leakage, the ELAV base demonstrated the ability to maintain its position on the simulated vaginal mucosa even after dilution. This finding suggests that the ELAV base may provide a longer residence time.

### 3.4. Self-Emulsification Characteristics

The self-emulsifying properties of the ELAV base demonstrated adaptability, allowing it to form emulsions effectively even with small volumes of VFS. This characteristic underscores its potential in enhancing drug delivery within the vaginal environment, which naturally experiences fluctuations in fluid levels (reduced vaginal fluid production, postmenopausal syndrome, etc.). The ability of the ELAV base to self-emulsify with limited fluid suggests that it may facilitate improved drug dispersion and adhesion to the mucosal surface, which are key factors in prolonged retention and therapeutic efficacy. The observed reduction in droplet size over time is an indicator of a robust emulsification process that can further enhance the bioavailability of encapsulated APIs. This dynamic adjustment in droplet formation allows the ELAV base to potentially deliver a more uniform and effective distribution of APIs, which may overcome limitations in spreadability or release as seen in traditional bases. The self-emulsifying properties of ELAV, as demonstrated in this fluorescence microscopy study, suggest that this base can adapt to varying vaginal fluid volumes and effectively encapsulate both hydrophilic and lipophilic APIs. The encapsulation of curcumin in the lipid droplets and the distribution of fluorescein sodium in the aqueous phase suggest that the ELAV base has the potential to release lipophilic APIs gradually while allowing for a faster release of hydrophilic compounds. This dual-release mechanism may offer more tailored and efficient treatment options, depending on the solubility and pharmacokinetics of the incorporated APIs.

## 4. Materials and Methods

### 4.1. Materials

This study was designed to comprehensively evaluate properties of the anhydrous vaginal base (trade name: Ellage^®^ Anhydrous Vaginal (PCCA, Houston, TX, USA)), hereinafter referred to as the ELAV base. ELAV is an off-white, shiny, smooth cream composed of Medium-Chain Triglycerides NF, Hard Fat NF, Polyoxyl Stearate NF, Glyceryl Monostearate NF, Poloxamer 407 NF, Glyceryl Ricinoleate, Polyoxyl 20, and Cetostearyl Ether NF. ELAV contains a self-emulsifying drug delivery system that creates a micro-emulsion when it comes in contact with water in vaginal fluid. This emulsion releases the APIs from the base to the mucosa. Once the APIs are released, the emulsifier system in the base also holds the drugs at the surface and increases the contact time. For comparisons of various methods, the following were used: a water-containing vaginal base (trade name: VersaBase^®^ Cream (PCCA, Houston, TX, USA)), hereinafter referred to as VBC; a compounding base for mucous membranes (trade name: MucoLox™ (PCCA, Houston, TX, USA)), hereinafter referred to as ML; and a topical, vaginal, and rectal gel base (trade name: VersaBase Gel), hereinafter referred to as VBG, as well as other OTC vaginal lubricants, moisturizers, and a Plasticized Compounding Base (trade name: PCCA Plasticized™ (PCCA, Houston, TX, USA)), hereinafter referred to as PCB. The vaginal bases, APIs, and compounded medications were prepared and provided by PCCA (Professional Compounding Centers of America, Houston, TX, USA): Ellage^®^ Anhydrous Vaginal (Lot 0527009), VersaBase^®^ Cream (Lot 9156966), PCCA Plasticized™ (Lot 8559865), OTC I vaginal lubricant (Replens™ Long-Lasting Vaginal Moisturizer (Church & Dwight Co., Ewing Township, NJ, USA)), OTC II vaginal lubricant (Replens™ Moisture Restore External Comfort Gel (Church & Dwight Co., Ewing Township, NJ, USA)), MucoLox™ (Lot 8917769), VersaBase Gel (Lot 8581476), and Gynol II Spermicide (Caldwell Consumer Health LLC, Madison, NJ, USA). The compounded medications assessed in this study were as follows: estriol 0.1% and testosterone 0.1% in ELAV; estriol 0.1% and testosterone 0.1% in VBC; amitriptyline 2% and baclofen 2% in ELAV; amitriptyline 2% and baclofen 2% in VBC.

### 4.2. Experimental Design

The development of the vaginal vehicle was guided by critical target attributes—anhydrous composition, non-irritative nature, mucoadhesive properties, and self-emulsifying behavior. These characteristics were systematically assessed through a combination of in vitro and ex vivo methods aimed at evaluating biocompatibility and functional performance. The overall experimental strategy is summarized in Figure S6.

### 4.3. Human Vaginal–Ectocervical Tissue Viability MTT Assay

This study employed the EpiVaginal™ VEC tissue model (MatTek Life Sciences, Ashland, MA, USA). Four test products were evaluated: ELAV compounding base, OTC I and OTC II vaginal lubricants, and Gynol II Spermicide. The VEC-100 cells were maintained in culture media in accordance with the manufacturer’s instructions until they were prepared for testing [22]. A volume of 100 μL of each product was applied to the tissue model in duplicate for 1, 4, 16, and 24 h, with an untreated set of tissues serving as a negative control and following the designated exposure periods. Following the exposure period, the test solutions were removed, and the 300 μL MTT solution (1 mg/mL) was applied to the basal side of the tissues. After a 3 h incubation period at 37 °C, the purple formazan product was extracted with extractant solution, and the optical density (OD) was measured at 570 nm with a reference wavelength of 650 nm using a CLARIOstar plate reader (BMG LABTECH GmbH, Ortenberg, Germany). The relative cell viability of each test product was calculated as a percentage in comparison to the negative control: % viability = OD (treated tissue)/OD (untreated tissue) × 100 [23].

### 4.4. Hen’s Egg Test-Chorioallantoic Membrane Assay

The irritation potential of the ELAV compounding base was evaluated in comparison to positive (0.1 N sodium hydroxide solution) and negative (0.9% sodium chloride solution) controls, following the ICCVAM HET-CAM recommended test method [25]. Fertilized hen eggs were incubated at 37 °C for 9 days to allow the development of the CAM. After the incubation period, a small window was carefully created in the eggshell with the help of a scalpel and tweezers to expose the CAM for testing. For each test, 0.5 mL of the respective formulation (ELAV base and controls) was applied. The membrane was observed for an initial 5 min period, and observations were extended up to 20 min to detect any delayed reactions. Each experiment was conducted in triplicate. Data collection involved recording the occurrence and intensity of lysis, hemorrhage, and coagulation, as well as the time required for each reaction. Photographic documentation was undertaken at the outset of the experiment (0 min), after 0.5 min, and at the conclusion of the assays (5 min). The irritation score (IS) was calculated based on the presence and intensity of lysis (vessel disintegration), hemorrhage (vessel bleeding), and coagulation (blood clotting). The products were classified as non-irritants with IS 0–4.9 or irritants with IS ≥ 5 [42].

### 4.5. Effect of the Base on the pH of HVF

The evaluation of the base’s effect on the pH of HVF was conducted in vitro using VFS following the methodology described below. Approximately 50 mL of VFS was heated to 37 °C, and the initial pH of the fluid was measured using a compact pH meter LAQUAtwin-pH-22 (Horiba Advanced Techno Co., Ltd., Kyoto, Japan). To 5.0 g of the ELAV base, VFS was added incrementally and mixed thoroughly. The VFS was added to the base in the following sequence to reach a final total volume of 10 mL, with pH measurements taken after each addition: 0.5 mL (2 times), 1 mL (4 times), and 2.5 mL (2 times). This procedure was repeated for 5.0 g of VBC, ML/VBG (50:50), and the OTC vaginal moisturizer. The VFS was prepared based on the composition proposed by Owen and Katz (1999) [36], and 1.0 L of this fluid contained the following: sodium chloride, 3.51 g; potassium hydroxide, 1.40 g; calcium hydroxide, 0.222 g; bovine serum albumin, 0.018 g; lactic acid, 2.00 g; acetic acid, 1.00 g; glycerol, 0.16 g; urea, 0.4 g; glucose, 5.0 g.

### 4.6. Evaluation of the Mucoadhesive Properties

#### 4.6.1. Ex Vivo Bioadhesion Testing Using a Texture Analyzer

In order to evaluate the mucoadhesive properties of the ELAV compounding base, the well-established combination ML/VBG (50:50) and an OTC vaginal moisturizer, which claims to provide long-lasting effects for up to 3 days, were selected as references for a comparative study.

Experimental Procedure: Frozen porcine vaginal tissues (BioIVT, Westbury, NY, USA) (Lot #: PIG15407–PIG15409) were thawed at room temperature, and the epithelial surface was excised and cut into circular samples of approximately 10 mm in diameter using a biopsy scalpel punch. The bioadhesive properties of the ELAV base, ML/VBG (50:50), and the OTC vaginal moisturizer were evaluated by premixing each formulation with VFS in a ratio of 3:1 (*v*/*v*) [36]. Then, 0.05 mL of each test sample in VFS was applied to a cellulose acetate membrane, which was fixed to the CTX Texture Analyzer (Ametek Brookfield, Middleborough, MA, USA) using a mucoadhesion rig.

The porcine epithelial tissue was attached to the cylinder probe of the texture analyzer using cyanoacrylate glue. The probe was lowered at a speed of 2.5 mm/min until it contacted the test product, applying a trigger force of 1 g, followed by an applied force of 60 g. After a 3 min hold time, the probe was raised at a speed of 2.5 mm/min, separating the epithelium from the test product. A total of six replicates were conducted for each formulation, along with a negative control (VFS alone). The experiments were performed at a constant temperature of 37 °C to simulate body conditions.

The work of adhesion (g·mm), representing the mucoadhesive force, was calculated as the area under the force versus displacement curve.

#### 4.6.2. Ex Vivo Testing on Porcine Vaginal Tissues

This test was conducted in accordance with the in vitro studies by Song et al. [48] and Cazorla-Luna et al. [49]. Frozen porcine vaginal tissues (BioIVT, Westbury, NY, USA) were used for an ex vivo study. The tissues were thawed at room temperature, cut into 2 × 2 cm pieces, and washed with Hanks’ balanced salt solution. Prior to the experiment, the tissues were equilibrated at 37 °C. Afterwards, the ex vivo vaginal tissues were fixed on stainless steel using cyanoacrylate glue and washed with the VFS [42].

Fluorescein sodium (Sigma-Aldrich, Burlington, MA, USA, F6377) (Lot SLBL5470V), a fluorescent dye, was dissolved in ethanol and added to the ELAV base, ML/VBG (50:50), and the OTC vaginal moisturizer to allow visualization of the mucoadhesion properties. Control groups of vaginal formulations without the fluorescent dye were also included. The test products were applied (50 μL/cm^2^) to the surface of the porcine vaginal tissues and evenly distributed using a pellet pestle. The test tissues were rinsed intermittently in VFS by immersing the plates at predetermined time points (5, 10, 15, 20, 25, 30, 40, 50, 60, 70, 80, and 90 min) to simulate vaginal conditions. Fluorescence was observed under UV light at 390 nm and captured using a smartphone camera (iPhone XII, Apple Inc., Cupertino, CA, USA).

### 4.7. Evaluation of the Leakage Potential

An adapted in vitro leakage test [27] was used, including VFS to mimic HVF with Petri dishes coated with agarose. Four samples were collected for analysis: ELAV base, estriol 0.1% and testosterone 0.1% in the ELAV base, amitriptyline 2% and baclofen 2% in the ELAV base, and an OTC long-acting vaginal moisturizer. Each sample was tested in both diluted and undiluted forms. Dilution was achieved by adding 0.75 mL of VFS to 2 mL of base or formulation, and mixing was carried out. To simulate a moist, vertical surface similar to vaginal mucosa, Petri dishes (100 mm) were prepared by filling each dish with 25 mL of a 1.5% (*w*/*v*) agarose solution in VFS, which was allowed to solidify at room temperature. Prior to the experiment, the agar plates were placed in an incubator Model 2020 (VWR™ International, Cornelius, OR, USA) at 37 °C for 1 h, positioned at a 60° angle. A 0.5 mL aliquot of each sample was applied in triplicate to the top of agar plates using a syringe. The migration distance of the test formulations along the agar plate was measured for 5 min.

### 4.8. Self-Emulsifying Properties

Light microscopy was conducted to observe the emulsifying dynamics of the ELAV base in comparison with PCB and VBC. The ELAV base, VBC, and PCB were first gently mixed with VFS in a 1:2 (*w*/*v*) ratio and incubated at 37 °C for 5 min (sample preparation). To assess droplet formation and size, each sample was observed under a light microscope. Then, 10 μL of VFS and 10 μL of the sample were placed on glass slides (1:1 ratio). The mixtures were placed with a pipette tip, incubated at 37 °C for 5 min, and covered with a coverslip for microscopic examination at 100× magnification (using a 10× objective and a 10× ocular lens). To evaluate the self-emulsifying properties of the ELAV base when containing APIs, amitriptyline 2% and baclofen 2% were incorporated into the ELAV base. To assess droplet formation under different conditions, the ELAV base was also mixed with VFS at ratios of 9:1, 3:1, and 1:1 and examined under a light microscope at 10× magnification.

Fluorescence microscopy was used to evaluate the distribution patterns of hydrophilic fluorescein sodium (Sigma-Aldrich, #F6377) and lipophilic curcumin (PCCA, #C30-3497) substances within the formulations and to compare them with the performance of the reference bases. Test formulations were prepared by adding 0.02% fluorescein sodium and 1% curcumin into the ELAV base, together with 5% glycerol as a cosolvent (sample preparation). The formulations were mixed for 2 min using an electronic mortar and pestle (EMP) mixer, GAKO UNGUATOR (GAKO International GmbH, Munich, Germany), which was set to a speed of 5 to achieve a homogeneous distribution. Then, a portion of each sample with fluorescein or curcumin was mixed with VFS in a 1:2 (*w*/*v*) ratio and incubated at 37 °C to simulate vaginal conditions. Within 5 min after preparation, samples of the test formulations, with and without VFS, were observed under a combination of white and green fluorescent light. Fluorescence microscopy tests were performed using a Nikon Eclipse TS100 inverted phase microscope equipped with NIS-Elements imaging software (version 5.02, Nikon, Tokyo, Japan) and a Lumencor^®^ MIRA Light Engine (4-NII-FA) (Lumencor, Inc., Beaverton, OR, USA). A filter set with excitation and emission spectra of 460–490 nm and 500–560 nm was used for fluorescence excitation, allowing detailed visualization of the self-emulsifying properties of the samples at 10× magnification.

## 5. Conclusions

This study confirms that the novel anhydrous ELAV base successfully embodies the key design properties essential for vaginal semisolid formulation: anhydrous composition, non-irritative nature, strong mucoadhesion, and effective self-emulsifying behavior. The ELAV base demonstrated a strong biocompatibility profile, showing no cytotoxicity or irritation in standardized assays (MTT and HET-CAM), and it maintained minimal impact on the physiological pH of VFS. Its strong mucoadhesive properties, confirmed through ex vivo testing and texture analysis, suggest enhanced retention time on vaginal mucosa, which is essential for prolonged therapeutic action. ELAV’s self-emulsifying capability allows efficient dispersion of both lipophilic and hydrophilic APIs even in low-fluid environments. Compared to water-containing bases such as VBC and ML/VBG (50:50), ELAV offers several functional properties that may be beneficial under specific formulation conditions: longer retention time and lower leakage potential; the ability to adapt to varying vaginal fluid levels; and efficient dispersion of both hydrophilic and lipophilic APIs. Moreover, its anhydrous nature is expected to minimize the risk of API hydrolytic degradation, and the stability of promising formulations is planned for further investigation. ELAV thus emerges as an option for compounded vaginal formulations that demand these specific functional characteristics.

## Data Availability

The original contributions presented in this study are included in the article/supplementary material. Further inquiries can be directed to the corresponding author.

## Supplementary Materials

The following supporting information can be downloaded at https://www.mdpi.com/article/10.3390/ph19040585/s1. Figure S1: Ex vivo porcine vaginal tissue immediately after exposure to ELAV base (t = 0 min). The image shows the initial state of the tissue prior to incubation or rinsing with vaginal fluid simulant (VFS). Figure S2: The result of mixing ELAV (1), VBC (2) and PCB (3) bases with vaginal fluid simulant (VFS) in a 1:2 (*w*/*v*). The images demonstrate differences in self-emulsifying behavior among the tested bases. Figure S3: Light microscopy (100×) showing droplet formation in VBC, PCB and ELAV bases before and after mixing with vaginal fluid simulant (VFS). Images illustrate droplet formation in ELAV base with and without incorporated APIs (amitriptyline 2% and baclofen 2%). Figure S4: Light microscopy (10×) showing droplet formation in the ELAV base after mixing with different proportions of vaginal fluid simulant (VFS) (9:1, 3:1, and 1:1 ratios). The images illustrate the effect of increasing aqueous phase on the self-emulsification process. Figure S5: Fluorescence microscopy of ELAV base mixed with vaginal fluid simulant (VFS) containing curcumin (lipophilic) and fluorescein sodium (hydrophilic). Images obtained under white and green fluorescent light illustrate the localization of lipophilic curcumin within emulsion droplets and hydrophilic fluorescein sodium in the surrounding aqueous phase. Arrows indicate the distinct distribution of both substances. Figure S6: Experimental design for the evaluation of the key performance characteristics of the anhydrous vaginal base. Table S1: Relative cell viability for ELAV base, OTC I, OTC II, and Gynol II over time. Table S2: Effect of various vaginal bases on the pH of vaginal fluid simulant (*n* = 3).

## Abbreviations

The following abbreviations are used in this manuscript:

API	Active Pharmaceutical Ingredient;
CAM	Chorioallantoic Membrane;
ELAV	Ellage® Anhydrous Vaginal Base;
ET50	Toxic Exposure Time;
HET	Hen’s Egg Test;
HVF	Human Vaginal Fluid;
ICCVAM	Interagency Coordinating Committee on the Validation of Alternative Methods;
IS	Irritation Score;
ML	MucoLox™ Compounding Base;
MTT	3-[4,5-dimethylthiazol-2-yl]-2,5-diphenyltetrazolium bromide;
OD	Optical Density;
OTC	Over the Counter;
PCB	Plasticized™ Compounding Base;
PCCA	Professional Compounding Centers of America;
VBC	VersaBase® Cream Compounding Base;
VBG	VersaBase Gel Vaginal and Rectal Gel Base;
VEC	Vaginal–Ectocervical;
VFS	Vaginal Fluid Simulant.
