# Supplementary material for: Comprehensive Evaluation of a Mucoadhesive Self-Emulsifying Anhydrous Base for Vaginal Drug Delivery
Source: Pharmaceuticals (Basel). 2026 Apr 7;19(4):585. doi: 10.3390/ph19040585 (PMC13118548; doi:10.3390/ph19040585)
Supplement: Supplementary file 1 [file pharmaceuticals-19-00585-s001.zip › pharmaceuticals-3739055-supplementary.pdf]

Supplementary material for:

Comprehensive Evaluation of a Mucoadhesive Self-Emulsifying Anhydrous Base for Vaginal Drug Delivery

Guiyun Song [1,*], Yi Liu [1], Kendice Ip [1], Ashley Shan [1], Christine Vu [1], Kateryna Khokhlova [2], Oleksandr Zdoryk [3], Maria Carvalho [1] and Daniel Banov [1]

[1] Professional Compounding Centers of America (PCCA), Houston, TX 77099, USA; yliu@pccarx.com (Y.L.); kip@pccarx.com (K.I.); ashan@pccarx.com (A.S.); cvu@pccarx.com (C.V.); mcarvalho@pccarx.com (M.C.); dbanov@pccarx.com (D.B.)

[2] Department of Technology of Drugs, National University of Pharmacy, H. Skovorody Str. 53, 61002 Kharkiv, Ukraine; kateryna_khokhlova@ukr.net

[3] Department of Pharmaceutical Technologies and Medicines Quality Assurance, Institute of the Professional Skills Improvement in the Field of Pharmacy, National University of Pharmacy, H. Skovorody Str. 53, 61002 Kharkiv, Ukraine; alekszpharm@gmail.com

* Correspondence: gsong@pccarx.com

2. Results

2.1. Human vaginal-ectocervical tissue viability MTT assay

Table S1. Relative cell viability for ELAV base, OTC I, OTC II, and Gynol II over time

Time (hrs)	**Relative cell viability** (mean, % ± SD)			
	**ELAV**	**OTC I**	**OTC II**	**Gynol II**
**0**	100.00±3.17	100.00±3.17	100.01±3.17	100.00±3.17
**1**	96.13±2.01	87.95±1.03	91.81±1.60	84.75±3.21
**4**	89.71±0.21	86.63±9.81	96.89±1.04	39.16±3.05
**16**	80.35±2.33	72.99±1.55	95.87±9.82	3.54±0.10
**24**	78.36±0.19	63.52±1.63	75.3±17.94	2.69±0.17

2.4. Evaluation of the Mucoadhesive Properties

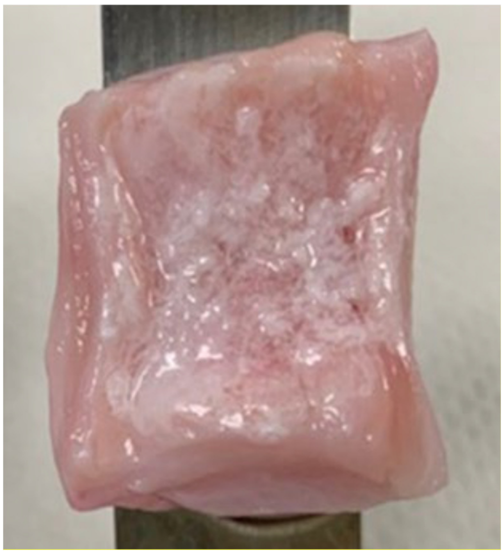

Figure S1. Ex vivo porcine vaginal tissue immediately after exposure to ELAV base (t= 0 min). The image shows the initial state of the tissue prior to incubation or rinsing with vaginal fluid simulant (VFS).

2.3. Effect of the base on the pH of HVF

Table S2. Effect of Various Vaginal Bases on the pH of Vaginal Fluid Simulant (n=3)

Volume of VFS, mL	ELAV		VBC		ML/VBG (50:50)		OTC moisturizer		VFS	
	**pH**	**SD**	**pH**	**SD**	**pH**	**SD**	**pH**	**SD**	**pH**	**SD**
**0.5**	4.65	0.02	4.94	0.01	4.68	0.08	4.69	0.05	4.54	0.06
**1**	4.54	0.04	4.54	0.01	4.58	0.06	4.64	0.12		
**2**	4.51	0.01	4.56	0.05	4.56	0.06	4.60	0.02		
**3**	4.52	0.01	4.67	0.10	4.54	0.04	4.71	0.02		
**4**	4.53	0.02	4.61	0.00	4.52	0.01	4.69	0.04		
**5**	4.53	0.03	4.60	0.02	4.51	0.01	4.62	0.00		
**7.5**	4.57	0.01	4.57	0.05	4.47	0.02	4.61	0.05		
**10**	4.60	0.01	4.55	0.05	4.51	0.01	4.62	0.06		

2.6 Self-emulsifying properties

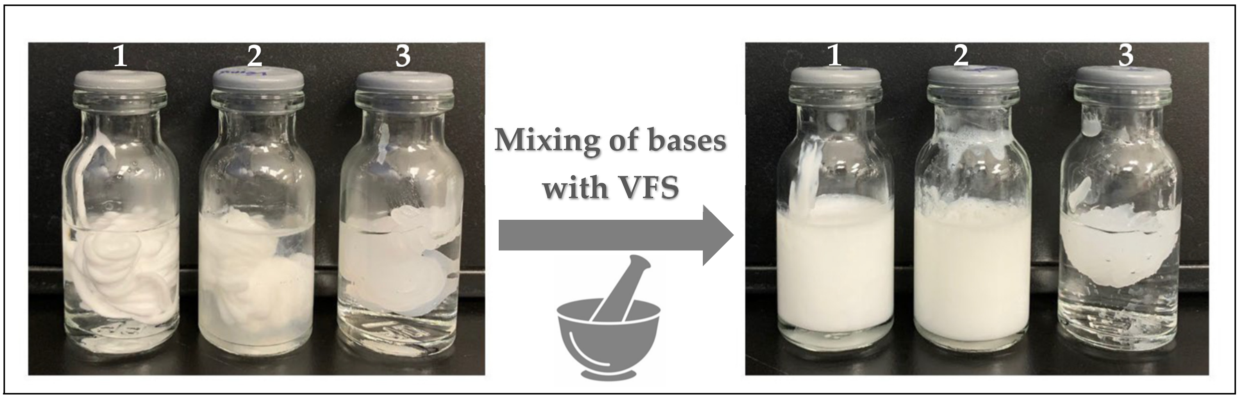

Figure S2. The result of mixing ELAV (1), VBC (2) and PCB (3) bases with vaginal fluid simulant (VFS) in a 1:2 (w/v). The images demonstrate differences in self-emulsifying behavior among the tested bases.

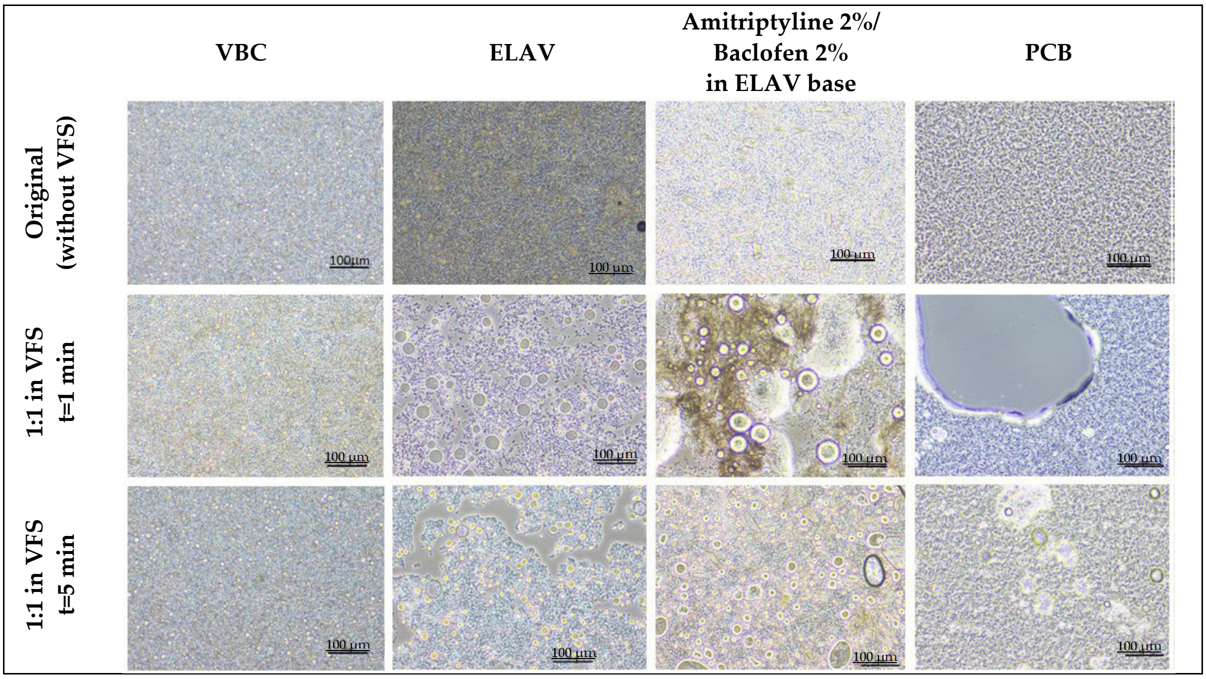

Figure S3. Light microscopy (100×) showing droplet formation in VBC, PCB and ELAV bases before and after mixing with vaginal fluid simulant (VFS). Images illustrate droplet formation in ELAV base with and without incorporated APIs (amitriptyline 2% and baclofen 2%)..

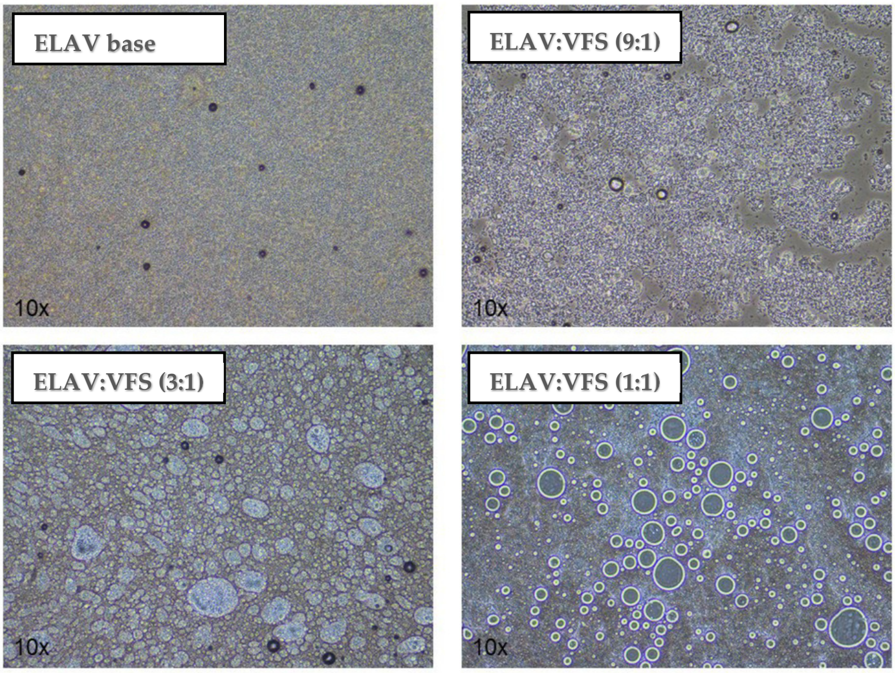

Figure S4. Light microscopy (10×) showing droplet formation in the ELAV base after mixing with different proportions of vaginal fluid simulant (VFS) (9:1, 3:1, and 1:1 ratios). The images illustrate the effect of increasing aqueous phase on the self-emulsification process.

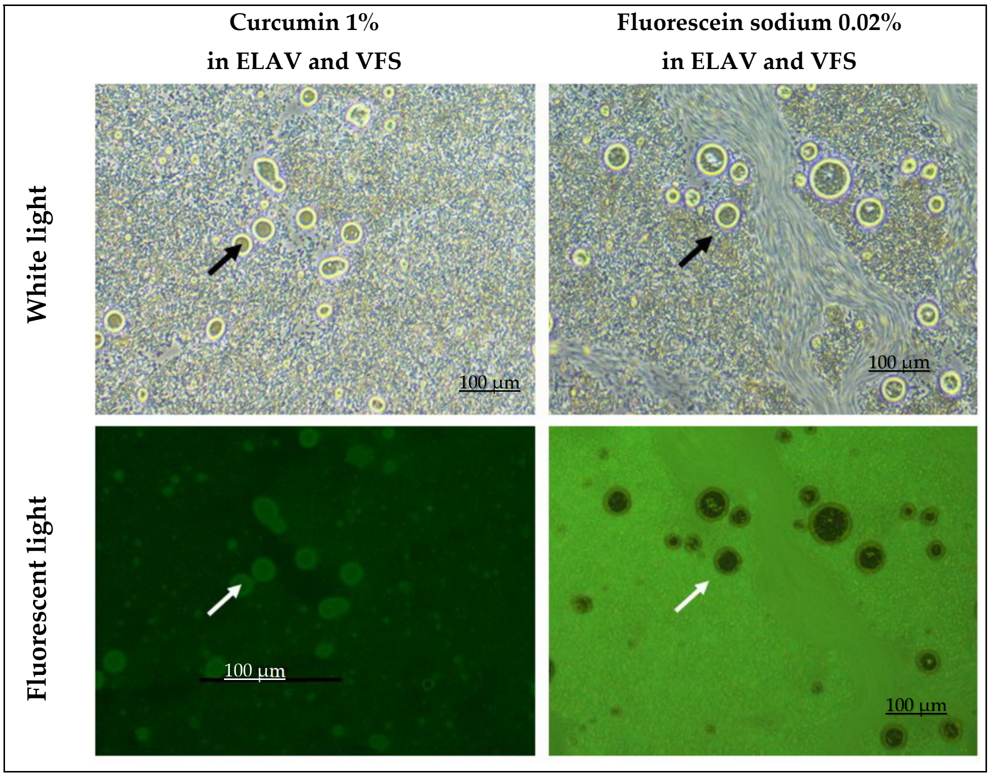

Figure S5. Fluorescence microscopy of ELAV base mixed with vaginal fluid simulant (VFS) containing curcumin (lipophilic) and fluorescein sodium (hydrophilic). Images obtained under white and green fluorescent light illustrate the localization of lipophilic curcumin within emulsion droplets and hydrophilic fluorescein sodium in the surrounding aqueous phase. Arrows indicate the distinct distribution of both substances.

4. Materials and Methods

4.2. Experimental Design

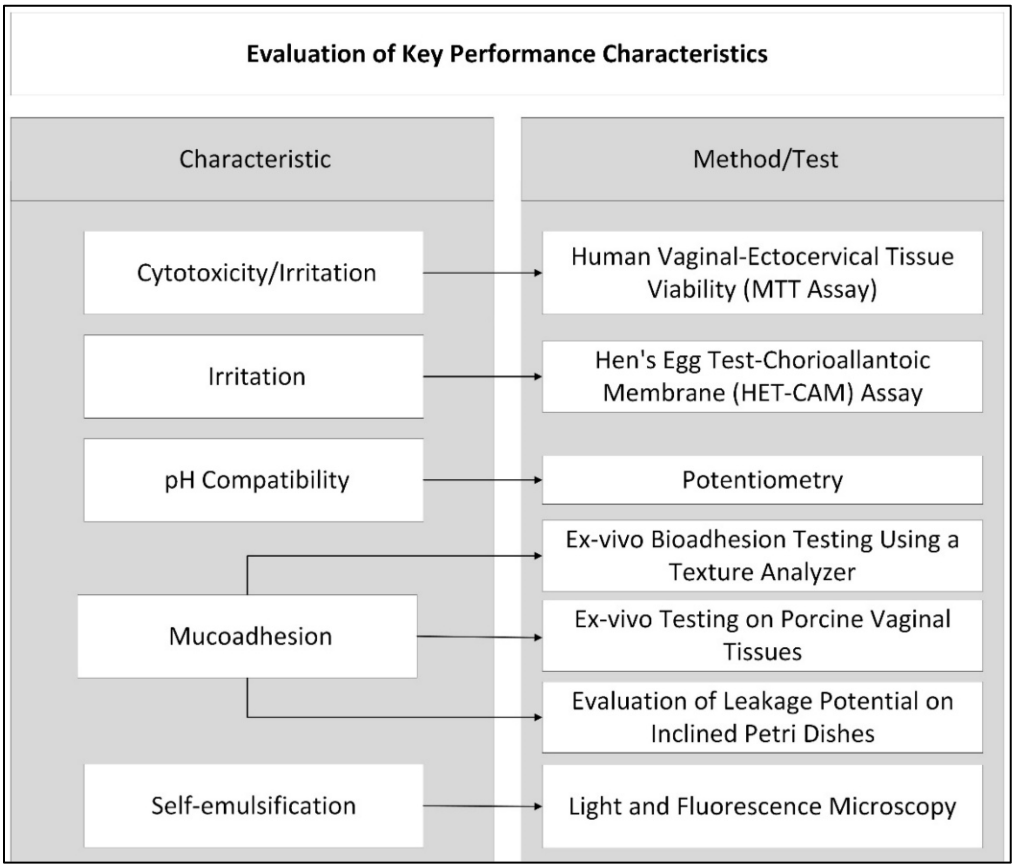

Figure S6. Experimental design for the evaluation of the key performance characteristics of the anhydrous vaginal base.
